# Plants Producing Ribosome-Inactivating Proteins in Traditional Medicine

**DOI:** 10.3390/molecules21111560

**Published:** 2016-11-18

**Authors:** Letizia Polito, Massimo Bortolotti, Stefania Maiello, Maria Giulia Battelli, Andrea Bolognesi

**Affiliations:** Department of Experimental, Diagnostic and Specialty Medicine-DIMES, Alma Mater Studiorum, University of Bologna, Via San Giacomo 14, 40126 Bologna, Italy; letizia.polito@unibo.it (L.P.); massimo.bortolotti2@unibo.it (M.B.); stefania.maiello2@unibo.it (S.M.); andrea.bolognesi@unibo.it (A.B.)

**Keywords:** ribosome-inactivating proteins, plant toxins, anticancer drugs, folk medicine, ethnopharmacology, ancient Egypt medicine, classic age medicine, traditional Chinese medicine, Ayurveda medicine, Unani medicine

## Abstract

Ribosome-inactivating proteins (RIPs) are enzymes that deadenylate nucleic acids and are broadly distributed in the plant kingdom. Many plants that contain RIPs are listed in the pharmacopoeias of folk medicine all over the world, mostly because of their toxicity. This review analyses the position occupied in traditional medicine by plants from which RIPs have been isolated. The overview starts from the antique age of the Mediterranean area with ancient Egypt, followed by the Greek and Roman classic period. Then, the ancient oriental civilizations of China and India are evaluated. More recently, Unani medicine and European folk medicine are examined. Finally, the African and American folk medicines are taken into consideration. In conclusion, a list of RIP-expressing plants, which have been used in folk medicine, is provided with the geographical distribution and the prescriptions that are recommended by traditional healers. Some final considerations are provided on the present utilization of such herbal treatments, both in developing and developed countries, often in the absence of scientific validation. The most promising prospect for the medicinal use of RIP-expressing plants is the conjugation of purified RIPs to antibodies that recognise tumour antigens for cancer therapy.

## 1. Introduction

Toxic substances, coming from either the mineral, vegetal or animal kingdom, have been frequently used for medical purposes since the earliest times. In particular, traditional medicine is based primarily on the use of plants, often taking advantage of those that are known for their toxicity, as a source of remedies for diseases that have always plagued humankind throughout the world. In addition, the knowledge of adverse events that arise from the consumption of some part of such toxic plants has its roots in very ancient time periods.

This review is dedicated to the role in traditional medicine played by plants from which ribosome-inactivating proteins (RIPs) have been extracted. RIPs are enzymes that depurinate nucleic acids and are widely distributed in the plant kingdom and beyond, whose biological role is yet to be elucidated. RIPs can be divided into two main categories: type 1, consisting of a single enzymatically active polypeptide, and type 2, consisting of a polypeptide that is similar to the type 1 RIP and has a lectin chain that is held together by a disulfide bridge (reviewed in [1]).

Some of the type 2 RIPs are highly toxic and for this reason have been recognized since ancient times by traditional healers, starting from *Ricinus communis* L. (castor bean), which produces ricin, the best known RIP and also one of the oldest known RIPs. A list of RIP-expressing plants, traditionally identified as medicinal plants for their pharmacological properties, is given in Table 1.

Plants that have historically been a source for traditional medical treatments have been recorded in documents that can be traced back over millennia to Western and Oriental ancient civilisations, starting from the Mesopotamian clay tablets in cuneiform (approximately 2600 BC). In other geographical areas, written documents are not available and the botanical knowledge that has been the foundation for the folk medicine is based on oral tradition.

## 2. Ancient Egyptian Medicine

A lot of information on the rich pharmacopoeia of ancient Egyptian civilization can be obtained from the papyri that were discovered in pyramids and tombs; hundreds of different compounds, many of which have not yet been identified, are described and recommended for several diseases. The identified products are minerals, vegetables and animals or human derivatives.

Castor bean seeds have been employed since ancient times for various purposes and have been found in Egyptian tombs that are approximately 6000 years old, probably because an oil can be extracted from the castor bean seeds and can be used as fuel for lighting lamps.

The pulp and oil of the castor bean fruit were utilized to prepare an ointment with medicinal or cosmetic purposes, as a remedy against abscessual illness or to favour hair growth, respectively. The first written record of these utilizations in ancient Egypt can be found in the “Ebers Papyrus”, a medical treatise that dates back to approximately 1500 BC, although it is considered to be a collection of much older different manuscripts. The “Ebers Papyrus” has over 800 recipes and gives us the most information about drug preparations. In the papyrus, an important role was given to the castor bean plant, in fact an entire section is devoted to castor bean [37].

Castor bean and *Bryonia dioica* Jacq. (bryony) roots were ingredients of a concoction described in “Ebers Papyrus” for the correction of polyuria of a child perhaps affected by diabetes [38]. Castor bean was also prescribed as a drug for internal use due to laxative or abortifacient intents. The toxicity of castor bean was well known, and only small amounts of seeds were utilized to prepare drugs for oral ingestion. In most cases, castor bean-containing medicinal preparations were based on the nontoxic fat component of the kernels, which was used in formulations both for external use with the aim of healing or soothing the skin and for internal use with a laxative purpose as castor bean oil.

Another plant that was mentioned in different Egyptian papyri is *Citrullus colocynthis* L. (colocynth). The fruit of the colocynth showed strong cathartic properties when taken boiled in water. This preparation was also able to induce menses and was used as an abortifacient. Additionally, in the “Ebers Papyrus”, a vaginal application of a colocynth-containing mixture was suggested to interrupt pregnancy. Moreover, the colocynth was reduced to powder and mixed with other ingredients to produce ointments for external use against parasitic worms.

In addition to castor bean, bryony and colocynth, other plants have been described in the “Ebers Papyrus” and in other Egyptian medical papyri because of their medical applications, possibly corresponding to *Hordeum vulgare* L. (barley), *Lagenaria siceraria* Stand. (bottle gourd), *Pisum sativum* L. (peas) and *Triticum aestivum* L. (wheat) [155], which are known to express RIPs ([156], reviewed in [157]). Barley, peas and wheat were prescribed for external application such as poultices against skin irritation and to obtain an astringent effect. The plants belonging to the Cucurbitaceae family were included in most preparations with plant ingredients probably due to their purgative action [155]. Barley was also used to detect pregnancy and to determine the sex of the foetus (reviewed in [158]).

The biblical plant *kikaion*, which is mentioned in the book of Jonah because it granted shadow to the prophet in the desert close to Ninive, was thought to correspond to the castor bean tree [159]. A great portion of the medical and botanical knowledge of the ancient Hebrew people, including the pharmacological properties of the plants, was possibly acquired during the period of permanence in Egypt [39].

## 3. Classic Age Medicine

Egyptian information about herbal medicine and drug preparation provided the base for the Greek and Roman pharmacopoeias, as confirmed in particular by Hippocrates (470–377 BC) in the “Hippocratic Corpus”, Celsus (25 BC–50 AD) in the “De Medicina”, Pedanius Dioscorides (40–80 AD) in the “De Materia Medica”, Pliny the Elder (23–79 AD) in the “Natural History” and Galeno (129–200 AD) in the “Galenic Corpus”.

Many plants were described by Hippocrates, who is considered the father of Western medicine, for therapeutic use in several diseases. Among them, there were various RIP-producing plants, such as *Momordica balsamina* L. (balsam), barley, bottle gourd, bryony, colocynth, *Sambucus nigra* L. (elderberry), *Viscum album* L. (mistletoe), castor bean, *Saponaria officinalis* L. (soapwort) and wheat [35,36].

Hippocrates prescribed the juice of the Cucurbitaceae or castor bean oil for laxative and detoxifying action, as well as soapwort pessaries to stimulate or increase menstrual flow, whereas barley could give strength and health. Wheat flour, sometimes as an alternative to barley, was an ingredient in gynaecological recipes for the preparation of tea and meal with the purpose of promoting fertility [36]. Elderberry was defined as “*nature's medicine chest*” by Hippocrates because of its health benefits in a variety of diseases, in particular as a diuretic and a laxative. Dioscorides first distinguished the elderberry from the dwarf elder (*Sambucus ebulus* L.), while recognizing that both plants exhibited the same medicinal properties. Today elderberry is still acknowledged for its properties against oxidative stress and its use is suggested for the treatment of influenza [126].

Mistletoe was recommended by Hippocrates as an emetic and for the treatment of spleen diseases and disorders associated with menstruation, whereas mistletoe-containing mixtures were recommended by Celsus for external use to treat abscesses, carcinoids, and scrofula. In a Celtic ritual ceremony mistletoe played a pivotal role because the Celtic druids believed that “*it given in drink will pass on fertility to any animal that is sterile and that it is an antidote to all poisons*”, as described by Pliny the Elder in the “Natural History”.

Dioscorides was a military physician and pharmacognosist of Nero’s Army; he is considered the father of pharmacognosy. He described more than 600 drugs of plant origin. About the application of castor bean, he reported that “*Thirty grains, cleaned, pounded into small pieces, and taken in a drink drives out phlegm, bile, and water through the bowels. They also induce vomiting, but this purging is harsh and extremely drastic, overturning the stomach excessively. Pounded and applied it cleans varicose veins and sunburn*” [21]. Accordingly, Pliny recognized the therapeutic properties as an aperient, which was attributed by the Greeks to castor beans.

The purgative and cathartic nature of bryony was known to Dioscorides and was also later prescribed by Galeno. Pliny believed that eating barley was a valid remedy against gastric ulcers. *Beta vulgaris* L. (beet) was acknowledged by the Romans as a laxative and the beetroot juice as an aphrodisiac. *Agrostemma githago* L. (corncockle) has been used to cure skin outgrowths, such as warts and tumours. *Cucurbita pepo* L. (pumpkin) was mentioned by Hippocrates, Dioscorides, Pliny and Galeno as a remedy against swelling. Dioscorides recommended the use of extracts of *Asparagus officinalis* L. (asparagus) root for the treatment of jaundice, sciatica and urinary and kidney dysfunctions. About the *Oryza sativa* L. (rice), Dioscorides says that “*it sustains poorly and tightens the belly*”. These antique manuscripts addressed the medical thinking of European physicians until the development of modern medicine, together with the oral traditions passed on by local folk medicine.

## 4. Traditional Chinese Medicine

The earliest Chinese pharmacopoeia has been attributed to Shennong, i.e., the Divine Farmer, a mythical ancestor of the Han population, who possibly lived in China in the third millennium BC. He would have begun a prehistoric oral tradition about agriculture and medicinal plants that was recorded into a lost compilation written in the first century BC. The subject was reworked and amplified by Li Shizhen, a sixteenth-century Chinese doctor who wrote the “Compendium of Materia Medica”, a medical treatise that included the classification of the herbs to be used as medications for treating diseases. The currently available text, “The Divine Farmer’s Materia Medica” [28], is a result of a series of rearrangements carried out over the centuries. Traditional Chinese medicine includes among herbal drugs a number of plants that produce RIPs, as detailed in Table 1.

Fruits and roots of the Cucurbitaceae were used: (i) to treat dehydration that was caused by fever or great heat; (ii) for mucolytic action in individuals with respiratory infections; (iii) to resolve intestinal constipation due to dry stools; and (iv) to induce menstruation, the expulsion of a retained placenta and abortion ([28], reviewed in [29]). The latex of the castor bean plant was instilled in the ear of the patient to cure rhinitis, whereas the seeds were used for anthelmintic activity. For external use, a seed poultice or leaf juice was recommended to treat ulcers and chronic wounds (reviewed in [112]). Leaves, inflorescences and seeds of *Celosia argentea* L. (cockscomb) have been prescribed for internal use to cure haematological and gynaecologic disorders and for external use in the treatment of inflammation and as a disinfectant [42].

*Phytolacca acinosa* Roxb. (Indian poke) and *Phytolacca americana* L. (pokeweed) roots were given both for internal use, in the case of constipation to induce a laxative effect or as a diuretic in the case of anasarca and ascites, and for external use to reduce swelling and nodules. Both in Chinese and Unani traditional medicines, the *Cinnamomum camphora* L. (camphorwood) was utilized to alleviate pain, in particular toothache, and also as a source of a topical application to treat sores and boils, whereas the seeds of purging croton were indicated to reduce oedema and ascites (reviewed in [46]).

According to traditional medicine in China, India and other oriental countries, *Clerodendrum inerme* L. (garden quinine) has been used for the cure of inflammatory diseases, including arthritis and rheumatic pain, skin diseases and fever and for the treatment of cancer [59]. The fruits of the colocynth have been prescribed for the treatment of gastrointestinal diseases, respiratory infections, diabetes and cancer (reviewed in [53]). Fresh *Stellaria media* L. (chickweed) juice has been administered for the treatment of viral diseases and dermatitis [132]. A variety of plants that were used in traditional medicine in China, India, Japan and Korea to treat diabetes due to their anti-hyperglycaemic activity includes *Trichosanthes cucumeroides* Maxim. (Japanese angelica-tree), barley, *Zea mays* L. (corn), asparagus, *Panax ginseng* Meyer (Chinese ginseng), *Panax quinquefolium* L. (American ginseng) and a number of Cucurbitaceae (reviewed in [23]).

The folk medicine of other Southeast Asian countries exploits the pharmacological properties of the local flora; in Malaysia, the leaves, stem-bark and stem-wood of *Cinnamomum porrectum* (Roxb.) possess antibacterial activity and have been traditionally used to treat wound infections [49].

Folk herbal medicine is still diffused in a large proportion of the population, mostly in developing countries for cultural reasons, and exists side by side with modern medicine. This is true for traditional Chinese medicine in China and also in Japan where this system of traditional healing survives under the name of Kampo medicine and was first codified in the tenth century by Yasuyori Tamba in the “Ishimpo” [160].

## 5. Ayurveda Medicine

The oldest known Hindu text on life sciences and Ayurveda folk medicine is “Charaka Samhita or Compendium of Charaka” and was written in Sanskrit. Charaka, a physician who lived in the second century BC, reported the teachings of a tradition that dated back to 1000 BC and was possibly based on the much more ancient Vedic wisdom. The current version of the manuscript, which was revised approximately 1600 years ago, discusses a variety of medical subjects. It also contains much botanical information and a series of instructions to prepare therapeutic formulations [161]. A number of RIP-expressing plants are included in such recipes in addition to the antidiabetic herbs and garden quinine, which were already mentioned in relation to traditional Chinese medicine, as described below.

Different parts of *Abrus precatorius* L., commonly known as Indian liquorice or jequirity, have been used both in Ayurvedic and Tamil folk medicine, including the seeds, leaves and roots, to prepare oil, tea or concoctions. Traditional healers knew about the poisonous effects of the ingestion of the seeds; therefore, they followed various methods of purification to abolish or at least mitigate the toxicity, such as boiling the seeds in milk before they were administered to patients. The plant has been claimed to possess aphrodisiac and hair-growth promoting activities. It was used to treat urticaria, migraine, malaria, tetanus and conjunctivitis due to its anti-pruritic, analgesic, anti-pyretic, anti-spasmodic and anti-phlogistic properties, respectively. The use of jequirity was also recognized to produce therapeutic effects including anti-tumour, expectorant, purgative, anti-diabetic, abortifacient, anthelmintic and anti-bacterial effects, in particular against ulcers and wounds. After being boiled, the seeds can be safely eaten in a similar fashion as nutritious beans ([13], reviewed in [162]).

Seeds, roots and leaves of the castor bean plant, called “erandah” in Sanskrit, have been used in Ayurveda as well as in Siddha medicine of the Tamil Nadu in South India and Sri Lanka for different purposes, including as a decoction for internal use or a poultice for external application, often as a polyherbal formulation. The castor bean tree has been credited to possess analgesic, anti-inflammatory and antipyretic properties, thus offering relief for every type of arthritic pain, swelling and feverishness. It was also used in digestive disorders and as an anthelmintic, anti-tumour, anti-convulsive, anti-bacterial and anti-diabetic agent. Castor bean oil was known to favour hair growth and to have aphrodisiac, cathartic and labour-inducing actions (reviewed in [113]).

Among the plants of the Ayurveda tradition that were present in preparations due to their therapeutic actions, *Momordica charantia* L. (bitter melon) has been used for thousands of years. The fruit, also identified as karela, is also harvested as food. Every part of the plant possesses pharmacological action, including the seeds, roots, leaves and particularly the unripe fruits, which have anti-mutagenic activity, are able to scavenge superoxide and hydroxyl radicals and are known for their anti-diabetic activity (reviewed in [93]). In ancient times, the juice of the fruit was used as a remedy in cases of snake poisoning. It was also considered effective for the alleviation of pain in the joints and against chronic fever. It was deemed to be helpful in cases of jaundice and disorders of the liver or the digestive system for its diuretic, laxative and anti-helminthic actions. The juice of the leaves and fruits was applied externally to cure chronic disorders of the skin as well as in the treatment of burns, boils and eruptions. The ingestion of the plant may help control excessive sugar in the urine and blood, and its medicinal chemistry has been revised together with the biochemical properties and mechanisms of its beneficial effects in the treatment of diabetes (reviewed in [94,95]). A variety of herbal remedies were prescribed in Ayurvedic, Siddha and Unani medicine for the treatment of diabetes, including asparagus, bitter melon, castor bean, colocynth and jequirity. A decoction of cockscomb seeds has been reported to possess anti-diabetic properties, according to traditional Indian medicine [40].

*Abelmoschus esculentus* L. (okra) is cultivated in Africa, America, Asia and Southern Europe for its nutritional value. According to Ayurvedic medicine, the consumption of okra has been suggested for the management of various diseases such as gastric ulcer, diabetes and cancer [5]. *Trichosanthes cucumerina* L. (snake gourd) is an edible vegetable that is grown in Asia, Australia and the Pacific Islands, known since ancient times, and is also used in Ayurvedic, Siddha and Unani traditional medicines as a drug with anti-inflammatory, anti-cancer, anti-diabetic, anti-ovulatory, larvicidal, and hepato- and gastro-protective activity (reviewed in [133]). The ingestion of the fresh juice of the winter melon, also called kundur fruit, was suggested in Ayurveda for the treatment of diabetes, nervous diseases, peptic ulcers and urinary disorders, whereas in Korean traditional medicine, it was used to cure dropsy, liver and intestinal diseases (reviewed in [30]). In addition, a number of other RIP-expressing plants listed in Table 1 have been recognized as having anti-diabetic properties in Ayurvedic, Siddha and Unani medicine, although the mechanism of anti-diabetic activity has been attributed to plant components other than RIPs [54].

Ayurvedic medicine is still popular in India, and this care system is spreading around the world, not only as an alternative disease treatment but also as a philosophy for health maintenance.

## 6. Unani Medicine

The Greek and Roman knowledge about the therapeutic use of plants was absorbed by the Arabs, in particular Avicenna (980-1037 AD), who was the author of the “Canon of Medicine”, and was the starting point of Unani medicine, which was practiced in the Indian medieval Muslim culture. Thus, the crude traditional drugs of the ancient Egyptians became the foundation of the empirical *materia medica* until the 18th century. For instance, in Unani medicine, barley has been prescribed for many diseases and to soothe the bowels. In addition, barley water was considered effective in the treatment of fever.

During the Islamic Golden Age, Arab physicians became aware of the medical knowledge of India and China and established a connection between Oriental and Western medicines of ancient origin, as suggested by the fact that some of the plants that are described in Islamic medical treatises show similar clinical indications in both traditional Chinese and Unani medicine (reviewed in [46]). In addition, in Unani medicine, many herbal remedies were common to Ayurveda, in particular those reported for the treatment of diabetes. 

Unani medicine persisted in all areas of Islamic culture along the old Silk Road starting from Turkey, the Middle East, Persia and Pakistan, as well as in North Africa, although with some differences due to the variety of the local flora and folk traditions.

In various traditional medicines of the Middle East Unani area, including Jordan [50], Iran (reviewed in [51]) and Pakistan [22], the fruits, roots and seeds of the colocynth have been prescribed to cure diabetes and parasitic, infectious and inflammatory diseases but also as a diuretic, cathartic and abortive drug. In Jordanian traditional medicine, beet leaves and roots have been used to obtain relief against acute and chronic inflammation [34]. Rice has been identified as a remedy against diarrhoea in the traditional medicine of Palestine [100].

In traditional Iranian medicine, several edible fruits have been recommended for the management of peptic ulcers such as pumpkin and rice for their anti-acid and anti-oxidant activities (reviewed in [63]). The use of dwarf elder has been recommended as an anti-fungal remedy against mycotic skin infections [119] and as a painkiller to treat a variety of bone and joint pathologies [120]. In addition, the aqueous extract of castor bean leaves has been mentioned to alleviate the pain induced by chronic knee osteoarthritis [118].

In Pakistan traditional medicine, some uncommon uses are reported for balsam as a wound healer and peas as an anti-spermatogenesis agent [89]. *Amaranthus viridis* L. (amarant) has been used in Indian Ayurdeda and Pakistan traditional medicine as a whole-plant decoction to stop dysentery, the root juice has been used to treat inflammation, and the leaves were recommended for snake bites and scorpion stings [22]. *Chenopodium album* L. (lambsquarters) has been prescribed in Indian Ayurvedic and Pakistan Unani medicine under the name of bathu as a diuretic, laxative, sedative, and hepatoprotective drug, as well as in veterinary medicine for its anthelmintic properties [45]. The same use against animal parasites was traditionally diffuse in Ethiopia [44]. Poultices of leaves or seeds of castor bean were administered for external use to cure gout and rheumatism as well as to treat wounds, boils and sores, while the juice of the leaves was given orally to treat liver disease [22].

In Turkish folk medicine, beet leaves were recommended for the treatment of diabetes [32]; bottle gourd was used to treat headache by wrapping the head with its leaves, whereas the oil obtained from the ripe fruits of bitter melon, macerated in olive oil warmed by the sun, was combined with honey and used for the prevention and healing of gastric ulcers [80]. A decoction of dwarf elder flowers was used against cough, while an external application of a leaves decoction was considered effective in the treatment of snakebites and as a roots poultice to alleviate rheumatism pain. A decoction of elderberry flowers was suggested for a variety of pulmonary diseases including bronchitis and asthma. A decoction of mistletoe fruits and leaves was recommended for the treatment of many different affections [97]. This traditional medicine is still practiced among Islamic populations and many of the herbal remedies above listed are still recommended in Oriental and Middle East countries, as well as in Africa.

## 7. European Folk Medicine

The folk medicine of different European cultures was based on a mix of ancient knowledge and magic superstitions, primarily related to local plants that were growing in the temperate climate around the Mediterranean Sea or in North and East Europe. These old traditions were passed down orally for centuries, and no ancient written text is available.

According to Norse mythology, a mistletoe arrow caused the first winter by killing Odin’s son Balder, who was “the bright, the shining one” and who was also knowledgeable in healing herbs. The use of mistletoe extracts persisted in European folk medicine through the ages and is still part of alternative medicine for the complementary treatment of cancer, especially in the German-speaking area, under the name Iscador ([141], reviewed in [142]).

On the Mediterranean coasts of Europe, either castor bean leaves or the juice from the leaves was applied on the breast in the puerperium as galactogogue (reviewed in [112]). The ability of promoting lactation by castor bean may be interpreted as a result of the irritating action that can be induced by subtoxic amounts of ricin, but can be also caused by other undetermined substances present in castor bean leaves. Elderberry has been traditionally used as a decoction of flowers and leaves to treat cough and asthma, or for external application in the case of pimples and for eye inflammation, as well as an ointment that was prepared with bark and pith in olive oil to alleviate burns. Wheat preparations were applied on skin lesions as a bran decoction that was mixed with vinegar to cure sprains, as flour in olive oil in the case of whitlows, mixed with crushed snails for burns and wounds and as a whole germ in honey to cicatrize burns. A decoction of mistletoe was placed on chilblains to favour healing [124].

In Eastern Europe folk medicine, a decoction of elderberry flowers was recommended as a diuretic or for colds and influenza, as an antiseptic and an anti-catarrhal to reduce inflammation and treat fever. Many RIP-expressing plants have been used for their disinfectant and anti-inflammatory functions. Although the role of RIPs in the medicinal actions of these plants has not been ascertained, it is possible to hypothesize a contribution of RIPs for their antiviral activity, as well as because of macrophage sensitivity to their cytotoxicity (reviewed in [157]). The aerial part of mistletoe was mixed with garlic and was used as an ethanolic extract for internal use for sedative and diuretic properties in the case of nervous tachycardia and insomnia, arteriosclerosis and hypertension [127].

In Northern Europe, the elderberry has been used to prepare beverages either as a fresh berry juice or as a dried berry tea for the cathartic, diuretic and diaphoretic properties in the treatment of constipation, upper respiratory tract infections, back pain, headache and toothache (reviewed in [125]).

The herbal remedies of traditional folk medicine have become part of the natural products in the field of complementary and alternative medicine that today are utilized by a large number of European citizens.

## 8. African Folk Medicine

In Africa, treatment of diseases is mainly based on crude indigenous herbal products that are produced by adherence to the directions of traditional healers and have never been codified in medical treatises, but are solely based on oral tradition. Folk medicine has become enriched over the centuries and has been influenced by cultures, religions and migrations. Islamic medicine had an important influence, particularly in North Africa. This medicine is mainly based on preparations from the plant kingdom. The drugs are prepared by water extraction of the components from the whole plant or from single parts, either alone or more frequently in combination. The extracts are prepared in the form of decoctions, concoctions and infusions, for oral consumption, enemas and inhalations, or as ointments or pastes for topical applications. Among the plants that are used by African healers to prepare pharmacological remedies, there are some from which RIPs have been purified. 

The Passifloraceae *Adenia digitata* (Harv.) Engl. (modecca flower) and *Adenia volkensii* Harms (kilyambiti plant) are poisonous plants that are largely present in South and tropical Africa, respectively. Modecca flower roots, rubbed into swelling or applied warm, are used in the treatment of knee swelling, while a decoction is taken to treat skin ailments, leprosy, ulcers and swollen legs. A roasted rootstock powder of kilyambiti plant is used to treat coughs, colds, and pneumonia and is taken with milk to treat stomach ache and swelling. A leaf decoction is taken orally to treat bronchitis, coughs, and fever or is used as a purgative enema [20].

Balsam and bitter melon are used for the cure of various diseases and preparations containing every part of the plants, including fruits, leaves, roots, seeds and leaf juice or decoction are utilized both for internal and external treatment [88].

Castor bean crushed fruits or oil are used alone or in combination with the ashes or powder of various other plants for local application or is rubbed into the scarifications that are made on the ailing part of the body for the treatment of different ocular, cutaneous or articular affections [109]. The roots are chewed or boiled and drunk to induce uterine contraction and abortion [82].

Colocynth is used in Tunisia, Morocco and other Islamic Mediterranean countries. Different parts of the plant, especially fruits and seeds, are often used. Common preparations use fresh, warmed or dried plant material (often ground), as well as extracts that are used mostly in a liquid form. Extracts are prepared either in water or in aqueous mixtures that contain more lipophilic compounds (hot milk extractions, water/olive oil at various ratios) at a temperature that ranges from tepid to boiling. Ground plant material can be mixed with honey for ingestion or for a topical gynaecological application or with other plants for poultices. The main indications are for the treatment of diabetes, inflammatory disorders, dermatological problems and various type of infections. For centuries, the colocynth fruits have also been used for cancer treatment [53,56,57].

Various parts of *Jatropa curcas* L. (purging nut) are used in traditional African medicine to cure different diseases, from simple fevers to serious infectious diseases. Seeds are employed as purgative; leaves are taken to cure fever and rheumatism. The latex and stems are utilized as anti-inflammatory agents and are used to treat various infections and infestations. Root decoctions are consumed for gingivitis and toothache, eczema and skin parasites [73]. The stem bark is used for the treatment of infertility and miscarriage [74].

Jequirity roots are used as sedatives and anticonvulsants in Amerindian and African medicine. Extracts from the roots have a moderate sedative effect. Leaves that are given in association with palm oil are taken to treat convulsions [7].

The stalks of *Manihot esculenta* Crantz (tapioca), cut into 2–3-cm pieces and inserted into the vagina, are used as an abortifacient to increase the uterine contractive activity and for the expulsion of the foetus [82].

Mistletoe, given as the whole plant or milk infusion, is used to provide relief for painful menstruation, asthma, bronchitis, stomach ailments and to reduce tumour swellings [106]. Mistletoe leaves, given as a juice or as decoction, find application in the therapy of hypertension [143].

Pokeweed fruits, roots and leaves are traditionally used to treat wound, fibroid growth, swelling and rheumatism [106]. *Phytolacca dodecandra* L’Hér. (African soapberry) finds application in the treatment of malaria, rabies, sore throat, rheumatic pain and jaundice as well as for various skin disorders and diseases depending on common pathogens [108].

*Ximenia americana* L. (sea lemon) is included in the therapy of many different diseases. The most frequently used part of the plant is the root, but the leaves, bark, fruits, stem and seeds can also be used. The plant is given as decoction, powder or after maceration. The leaves are also used as aqueous crude extract. The main indications are for the treatment of burns and wounds, infections, inflammation, pain and gynaecological disorders. Sea lemon extracts are also used as a tonic, a laxative and as an antidote for food poisoning and snake/scorpion bites [149,150,151].

In Africa, the majority of the population still uses traditional medical systems for most or all of their health care. This occurs in particular in rural areas where modern health care facilities are scarce or difficult to reach; thus, most people still depend on folk medicine. 

## 9. American Folk Medicine

More than 50% of the species that belong to the plant kingdom are present in Central and South America. Over 3000 of these plants are used in folk medicine for the treatment of various ailments. 

Bitter melon is widely present in the Caribbean area, and it is administered as a leaf decoction or fruit juice for the treatment of diabetes. The leaf decoction is also used for the treatment of high blood pressure and various type of infections. Leaf baths are used for rheumatism therapy (reviewed in [18]).

Corn silk tea is consumed by Mexicans for oliguria and dysmenorrhea treatment, husk tea for amenorrhea, and seeds are used for flu, dysentery, obesity, as an antioxidant, and for hypertension (reviewed in [18]).

*Cucurbita maxima* Duchesne (autumn squash) and pumpkin seeds are used as a folk remedy by several South American populations to expel intestinal worms and to treat urinary ailments, irritable bladder and benign prostatic hyperplasia [62]. In the Caribbean, pumpkin, as a flower or fruit decoction, is taken for measles and small pox, and as leaf poultice, is applied to sprains (reviewed in [18]).

*Hura crepitans* L. (sandbox tree) latex is used by Amazonian people on ailing teeth to cause them to fall out painlessly and without bleeding. Other uses are as an antidote for snakebites, an emetic, a purgative and as a drug to cure rheumatism, elephantiasis and leprosy [72].

Jequirity leaves are administered in the Caribbean for fevers, cough, cold and consumption; castor bean leaf poultice is used for stomach aches, erysipelas, flu, and inflammation of the womb. In Brazil, castor bean oil is used for wound healing, burns, hair loss, purgative, and as an anthelmintic (reviewed in [18,19]).

*Mirabilis jalapa* L. (four o’clock flower) leaves, as a decoction or an infusion, are extensively used by many South American inhabitants for treatment of diarrhoea and inflammatory and painful diseases; by Mexican people, it is also employed as a laxative [85].

Purging nut latex is applied by Caribbean people to external wounds to provide significant cicatrizing activity. The leaves are given in different manners: a leaf bath is used for rash and sprains, leaf poultices for sores and pains and leaf decoctions for heat, diarrhoea and fever. In Peru, the plant is also known for its anti-leishmanial use ([75,76], reviewed in [18]).

Sea lemon bark, fruits and roots are used in Brazil to treat a series of disease, including diabetes [19].

Tapioca leaf juice, orally administered, is used in Nicaragua for the treatment of abdominal pain, as an anti-pyretic, for diarrhoea, aches and pains. In Papua New Guinea, baths with tapioca are advised for treatment of skin blemishes. In Peru, as poultice, it is used to treat wounds. In Colombia, it is also used in human adults as vermifuge [84].

In Central and Southern Americas, medicinal plants are part of the heritage of pre-Columbian cultures. To date, they often remain the first choice for consultation and treatment in these countries.

## 10. Modern Medicinal Use of Plant Producing Ribosome-Inactivating Proteins

Only in a few cases, the pharmacological effect of RIP-expressing plant was due to the presence of RIP whereas most of the effects can be attributed to other components. For example, the laxative action of castor bean, still exploited today, is due to the oily part of the seeds, which is devoid of ricin toxin. However, the purgative and anthelmintic actions attributed in various traditional medicines to many RIP-producing plant could be correlated with the irritative effect caused to intestine by the administration of RIPs, as reported in toxicological studies (reviewed in [163]).

In the case of both the abortive and antiviral effects of various Cucurbitaceae and other RIP-expressing plants, these medicinal activities have to be attributed to the presence of RIPs, as reviewed in [164,165], respectively.

In particular, trichosanthin is a type 1 RIP from Chinese snake gourd belonging to the Cucurbitaceae family that has been used in traditional Chinese medicine as abortifacient and is still used in Chinese clinics for inducing midterm abortion. Trichosanthin produces follicular atresia in mouse and causes the embryo fails to develop by eliciting death of syncytiotrophoblasts of placental villi in fertilized animals (reviewed in [137]). The mechanism has been related to its uptake by, and high toxicity to, placental trophoblast cells, that also justify its application in the treatment of choriocarcinoma [166].

Momordica Anti-HIV Protein (MAP30), α- and β-momorcharins are type 1 RIPs from bitter melon that have been used in the folk medicine of various countries. These RIPs have been studied for their broad anti-viral activities and are considered potential therapeutic agents in HSV and HIV infections. MAP30 is non-toxic to normal cells, while it is active against infection and replication of both HSV and HIV. Moreover, MAP30 in combination with other antiviral drugs improves the efficacy of anti-AIDS therapy, by inhibiting HIV replication in acutely and chronically infected cells (reviewed in [167]). 

Many medicinal plants have been claimed to possess anti-diabetic activity and are still used today for this reason in almost all the Oriental countries. Amongst these plants, the most studied is *Momordica charantia* L. and the active principles found in its fruit juice. However, its hypoglicemic activity, which has been observed in various animal experimentations and some clinical trials, is mainly due to an insulin-like protein unrelated to RIPs (reviewed in [95]).

In other cases, it is not yet clear whether the therapeutic activity that is ascribed to some plants is due to their ability to produce RIPs. A typical case is that of mistletoe and viscumin toxin, which is contained in the Iscador preparations that are marketed to fight cancer as an alternative and complementary medicine [141]. The anti-tumour activity of mistletoe preparation was evaluated through an overview of 23 clinical studies showing considerable heterogeneity of the trials and variable outcomes. Thus, properly designed further investigations are required to validate the efficacy of mistletoe as anti-cancer drug (reviewed in [142]). The anti-proliferative role of the toxic type 2 RIP present in mistletoe was not ascertained in these trials, however it cannot be excluded. Sometimes, great influence has been attributed to the growth conditions of the medicinal plant. Representative of this impact is the relevance of the plant on which mistletoe is a parasite on its pharmacologic properties (reviewed in [168]).

In addition, teas, liquid extracts, and capsules of dried flowers and berries of the European elderberry tree are currently suggested by integrative medicine to cure colds, constipation, fevers, flu and sinus infections, but no relation has been established with the RIP lectin contained in elderberry [169]. In vitro studies indicated anti-oxidant, anti-viral and anti-proliferative effects of elderberry fruit preparations. The aqueous elderberry extract showed promising results in the treatment of viral influenza infection of chimpanzees. An anti-viral and anti-inflammatory action has been suggested in humans, but need further support by more rigorous clinical trials. The pharmacological role of type 2 RIPs contained in these preparations has not been clarified, although their antiviral activity is known (reviewed in [125]).

The use of pumpkin as a remedy against swelling was known since the classic age. Indeed, its anti-inflammatory and anti-androgen activities may justify the modern application of pumpkin in the management of patients who are affected by benign prostatic hyperplasia (reviewed in [64]). However, no evidence is available that these effects may be related to the presence of a RIP. 

Some RIP-expressing herbal remedies such as the different varieties of ginseng are recommended by alternative, complementary and integrative medicine for the presence of various components other than RIP. 

A very new method to take advantage of the therapeutic effect of RIPs has been recently described. In a murine xenograft model, the gene of the type 1 RIP trichosanthin was delivered by a viral vector to human hepatocellular carcinoma tumour via intra-tumour injection, resulting in a significant inhibition of tumour growth [170].

## 11. Conclusions

Since the earliest times, humankind sought among the available herbs not only those that were edible for sustenance but also those that were suitable as remedies for diseases. The knowledge about the medicinal properties of plants was passed down orally long before being fixed in written texts, and only in recent times it has been subjected to a scientific validation to isolate the active components, check the toxicity, verify the effectiveness and understand the mechanism of action.

Folk medicine has always been based on the empirical wisdom of traditional healers. Not only it was, in the past, the unique resource against diseases, but it still represents the sole weapon available to fight illness for a large portion of the population in many parts of the world. Moreover, the need for new medicinal products addresses many studies towards the rich source of ethnobotany to discover pharmacologically useful compounds. 

In the Oriental countries, local folk medicine continues to be preferred over Western drugs in many cases because of the fidelity to a traditional life style. The Chinese National Ministry of Science and Technology supports multicenter randomized controlled studies carried out to evaluate the results of the clinical trials with traditional Chinese medicine. This traditional medicine is now recognized worldwide as effective in treating various diseases, including cancer, when used in conjunction with Western medicine, being able to reduce toxicity and side effects induced by chemotherapy and radiotherapy ([171], reviewed in [172]).

The interest in medicinal products of plant origin is increasing in the Western world because they are considered more “natural” and thus less toxic, or even because they are linked to a philosophy of life. This trend justifies the increased popularity and spread of Chinese and Ayurvedic medicine and homeopathy as well as of integrative medicine all over the world. The reason why many RIP-containing plants are reported in the pharmacopoeia of traditional medicine in large part is due to their toxicity. It is no coincidence that the word pharmaceutical derives from the Greek “pharmakon”, which means poison. Indeed, some of the required effects necessitate of a strong action, as in the case of purgative, abortifacient and anti-cancer drugs. The fact that some plants have been used in many countries, like for instance the castor bean, may depend on various causes, *in primis* the diffusion of a species in geographical areas with similar climate conditions, then the circulation of the traditional medicine in zones with economic and cultural exchanges. Considerable influence may have also had the written codification of the knowledge in texts that have been handed down over the centuries.

Medicinal effects, such as the anti-phlogistic, antalgic and anti-pyretic actions, are often attributed in traditional prescriptions to herbs that contain RIPs. Taking into account the high cytotoxicity of RIPs to macrophagic cells (reviewed in [173]), the presence of a RIP could contribute to the above activities by reducing the macrophagic response to pathogens, which is in many cases responsible of producing inflammatory cytokines. In addition, very often RIP-containing plants have been used in the treatment of various skin affections. Besides the anti-phlogistic effect, the presence of RIP in medicinal preparations for external use could have a disinfectant action and so promote healing of the lesions. However, the exact role of RIPs in herbal remedies remains unclear because in most cases, the traditional prescriptions consist of a mixture of different plants.

Today, the most promising prospects for the therapeutic use of plants that produce RIPs are related to the synthesis of conjugates, consisting of a purified RIP linked to a carrier, which should allow the specific binding to and the endocytosis of the resulting synthetic protein into the target cell to be deleted, such as cancer cells (reviewed in [157,174]).

This perspective was already envisaged by Ehrlich, who was dreaming of an ideal therapeutic agent made of a haptomer and an effectomer, that is a carrier able to specifically choose its target and a component with toxic properties able to kill the targeted cell. The discovery of monoclonal antibodies [175] provided highly specific haptomers. As far as the toxic component, the A chain of type 2 RIPs and the type 1 RIPs may represent the perfect effectomer, because they have a low toxicity unless being delivered inside the cell by a carrier. Thus, the so-called “immunotoxins”, obtained by linking a monoclonal antibody to a RIP, somewhat allow to realize the “magic bullet” idea of Ehrlich.

## Figures and Tables

**Table 1:** Ethno-pharmacological uses of RIP-expressing plants.

Plant Species, (Common Name), Family and RIP Found	Geographic Area	Therapeutic Application	Plant Tissue(s)	References
*Abelmoschus esculentus* (L.) Moench (okra) Malvaceae abelesculin	Africa	Used as nervine, stomachic stimulant Used for the treatment of gastritis, spasms	Fruits, seeds	[2,3]
	India	Used for the treatment of anaemia, cancer, diabetes, gastric ulcer, gonorrhoea, urethrorrhoea	Fruits	[4,5,6]
*Abrus precatorius* L. (jequirity) Fabaceae abrins	Africa	Used for the treatment of asthma, bronchitis, cold, complaints, convulsion, cough, epilepsy, fever, malaria, sexual asthenia, stomach cramps, tetanus, tuberculosis, urinary schistosomiasis, wounds	Leaves, roots, stems	[7,8,9,10,11,12]
	India, Southeast Asia	Used as abortifacient, analgesic, anthelminthic, anti-bacterial, anti-emetic, anti-pruritic, aphrodisiac, expectorant, hair growth-promoting, purgative, tonic Used for the treatment of amenorrhea, cancer, conjunctivitis, diabetes, diarrhoea, female fertility, fever, inflammation, intestinal problems, leucorrhoea, malaria, menstrual disorders, migraine, muscular skeletal disorders, postpartum complications, spasms, tetanus, urticaria	Fruits, leaves, roots, seeds	[9,13,14,15,16,17]
	Caribbean	Used for the treatment of cold, consumption, cough, fever	Leaves	[18,19]
*Adenia digitata* (Harv.) Engl. (modecca flower) Passifloraceae modeccins	South Africa	Used for the treatment of knee swellings, leprosy, skin ailment, swollen legs, ulcers	Roots	[20]
*Adenia volkensii* Harms (kilyambiti plant) Passifloraceae volkensin	Africa	Used for the treatment of bronchitis, coughs, colds, fever, pneumonia, purgative, stomach ache, swelling	Leaves, rootstock	[20]
*Agrostemma githago* L. (corncockle) Caryophyllaceae agrostins	Mediterranean area	Used for the treatment of skin outgrowths (warts, tumours)	Seeds	[21]
*Amaranthus viridis* L. (amarant) Amaranthaceae amaranthin	India, Pakistan	Used as diuretic, laxative Used for the treatment of dysentery, inflammation, scorpion sting, snakebites	Leaves, roots, whole plant	[22]
*Aralia elata* (Miq.) Seem. (japanese angelica-tree) Araliaceae aralin	China, India, Japan, Korea, Russia	Used as anti-arrhythmic, tonic Used for the treatment of arthritis, diabetes, gastritis, gastrospasm, hepatitis, hypertension, neurasthenia	Whole plant	[23,24,25,26,27]
*Asparagus officinalis* L. (asparagus) Asparagaceae asparins	China, India, Japan, Korea	Used as emmenagogue Used for the treatment of arthralgia, diabetes, headache, urinary stones	Flowers, leaves, seeds	[4,23]
	Mediterranean area	Used for the treatment of jaundice, sciatica, urinary and kidney dysfunctions	Roots	[21]
*Benincasa hispida* (Thunb.) Cogn. (winter melon) Cucurbitaceae benincasins, hispin	China, Korea	Used as abortifacient, diuretic, emmenagogue, laxative, mucolytic Used for the treatment of dehydration caused by fever or great heat, diabetes, dropsy, hypertension, inflammation, liver and intestinal diseases, retention of placenta	Fruits, roots, seeds, whole plant	[28,29,30,31]
	India	Used for the treatment of diabetes, nervous diseases, peptic ulcers, urinary disorders	Fruits, roots	[4,23]
*Beta vulgaris* L. (beet) Amaranthaceae beetins, betavulgin	Turkey	Used as diuretic Used for the treatment of diabetes, malaria	Leaves	[32,33]
	Jordan	Used for the treatment of inflammation	Leaves, roots	[34]
	Mediterranean area	Used as aphrodisiac, laxative	Leaves, roots	[21,35,36]
*Bryonia dioica* Jacq. (bryony) Cucurbitaceae bryodins	Ancient Egypt	Used for the treatment of polyuria	Roots	[37,38,39]
	Mediterranean area	Used as detoxifying, laxative	Fruits	[21,35,36]
*Celosia argentea* L. (cockscomb) Amaranthaceae CCPs	India	Used as anti-bacterial, aphrodisiac, diuretic Used for the treatment of cancer, diabetes, eruptions, fever, gonorrhoea, healing of wounds, inflammation, injuries, jaundice, metrorrhagia, mouth ulcers, skin sores, spasms, ulcers	Leaves, seeds	[4,40,41]
	China	Used as ailment for eyes and liver, disinfectant Used for the treatment of blood diseases, broken bones, dysentery, dysuria, haematological and gynaecologic disorders, inflammation, mouth sore	Inflorescences, leaves, seeds	[42,43]
*Chenopodium album* L. (lamb’s quarters) Amaranthaceae CAP30	Africa	Used as anti-helminthic	Leaves, stems	[44]
	India, Pakistan	Used as anti-parasitic, diuretic, hepato-protective, laxative, sedative	Whole plant	[45]
*Cinnamomum camphora* (L.) J. Presl (camphorwood) Lauraceae camphorins, cinnamomins	Persia	Used as anti-septic, suppressor of sexual behaviours Used for the treatment of boils, cold, sores, toothache	Seeds	[46,47]
	China, Korea	Used for the treatment of asthma, boils, bronchitis, chills, colds, diarrhoea, hysteria, indigestion, muscle pains, nervousness, neuralgia, rheumatism, sores, sprains, toothache	Not indicated	[46,48]
*Cinnamomum porrectum* (Roxb.) Kosterm. (yellow camphor) Lauraceae porrectin	Malaysia	Used for the treatment of wound infections	Leaves, stem bark, stem wood	[49]
*Citrullus colocynthis* (L.) Schrad (colocynth) Cucurbitaceae colocins	Iran, Jordan	Used as abortifacient, analgesic, anti-parasitic, cathartic, diuretic, hair growth-promoting, purgative Used for the treatment of arthritis, diabetes, epilepsy, infections, inflammation, rheumatism	Fruits, roots, seeds	[22,50,51,52]
	India, Pakistan, Sri Lanka	Used as anti-bacterial, laxative Used for the treatment of abdominal worms, cancer, diabetes, gas troubles, gastroenteritis, liver and respiratory diseases, toothache, tuberculosis	Fruits, roots	[22,53,54,55]
	Africa, China, Middle East	Used as abortifacient, analgesic, anthelminthic, anti-bacterial, anti-oxidant, carminative, emmenagogue, immunostimulator, laxative, mucolytic Used for the treatment of cancer, cold, colic pain, cough, dehydration caused by fever or great heat, diabetes, dysentery, gastroenteritis, gynaecological-, respiratory-, skin- and urinary infections, hypertension, indigestion, inflammation, retention of placenta, sore throat, syphilis, toothache, tuberculosis	Fruits, roots, seeds	[28,29,53,56,57,58]
	Ancient Egypt	Used as abortifacient, anthelmintic, cathartic, emmenagogue	Fruits	[37,38,39]
	Mediterranean area	Used as detoxifying, laxative	Fruits	[21,35,36]
*Clerodendrum inerme* (L.) Schltdl. (garden quinine) Lamiaceae CIPs	Australia, China, India, Southeast Asia	Used as anti-fungal, hepato-protective Used for the treatment of inflammation, cancer, fever, pain, skin diseases, viral infections	Leaves	[59,60]
*Croton tiglium* L. (purging croton) Euphorbiaceae crotins	China	Used as laxative Used for the treatment of abdominal pain, ascites, intestinal inflammation, oedema, peptic ulcer	Fruits, seeds	[46,61]
*Cucurbita maxima* Duchesne (autumn squash) Cucurbitaceae cucurmoschin	Caribbean	Used for the treatment of prostate problems	Fruits	[62]
*Cucurbita pepo* L. (pumpkin) Cucurbitaceae pepocin	Caribbean, Iran	Used for the treatment of enlarged prostate gland, irritable bladder, measles, peptic ulcer, small pox, sprains, worm infections	Flowers, fruits, seeds	[18,62,63]
	Mediterranean area	Used for the treatment of enlarged prostate gland, swelling	Fruits	[21,64]
*Gynostemma pentaphyllum* (Thunb.) Makino (jiaogulan) Cucurbitaceae gynostemmin	China	Used as abortifacient, anti-microbial, anti-proliferative, emmenagogue, immune-stimulator, regulator of liver functions, laxative, mucolytic Used for the treatment of cancer, cardiovascular and renal diseases, dehydration caused by fever or great heat, diabetes, hyperlipoproteinemia, hypercholesterolemia, inflammation, retention of placenta	Fruits, leaves, roots	[23,28,29,65,66,67,68,69]
*Gypsophila elegans* M.Bieb. (annual baby’s breath) Caryophyllaceae gypsophilin	China, India	Used for the treatment of immune disorders, liver diseases	Not indicated	[70]
*Hordeum vulgare* L. (barley) Poaceae JIP60, barley toxins	China, India, Japan, Korea	Used for the treatment of cardiovascular diseases, diabetes, hyperuricemia, urinary stones	Seeds	[23,54,71]
	Ancient Egypt	Used as astringent and for the treatment of skin irritation	Seeds	[37,38,39]
	Mediterranean area	Used as tonic Used for the treatment of gastric ulcers, gynaecological disorders	Seeds	[21,35,36]
*Hura crepitans* L. (sandbox tree) Euphorbiaceae H. crepitans RIPs, crepitin, hurin	South America	Used as antidote for snakebites, emetic, purgative Used for the treatment of elephantiasis, leprosy, rheumatism, toothache	Bark, latex, seeds	[72]
*Jatropha curcas* L. (purging nut) Euphorbiaceae curcins, Jc-SCRIP	Africa	Used as purgative Used for the treatment of dysentery, eczema, fever, gingivitis, guinea worm sores, infertility, inflammation, jaundice, joint rheumatisms, miscarriage, mouth infections, skin parasites, syphilis, toothache	Bark, latex, leaves, roots, seeds, stems	[73,74]
	South America	Used for the treatment of diarrhoea, external wounds, fever, heat, leishmaniasis, rashes, sores, sprains	Bark, fruits, latex, leaves, roots	[18,75,76]
*Lagenaria siceraria* (Molina) Standl. (bottle gourd) Cucurbitaceae lagenin	India	Used as aphrodisiac, antidote, diuretic, tonic Used for the treatment of asthma, bronchial disorders, cough, diabetes, fever, hypertension, liver diseases, pain, ulcers, weight loss	Fruits	[77,78,79]
	Turkey	Used for the treatment of headache	Leaves	[80]
	Mediterranean area	Used as detoxifying, laxative	Fruits	[21,35,36]
*Luffa acutangula* (L.) Roxb. (ridged luffa) Cucurbitaceae luffaculins, luffangulin	India	Used for the treatment of diabetes	Fruits	[54]
*Luffa cylindrica* (L.) M.Roem (sponge gourd) Cucurbitaceae LRIP, luffins, luffacylin	India, Korea	Used for the treatment of diabetes, fever, haemorrhage, phlegm	Fruits	[54,81]
*Manihot esculenta* Crantz. (tapioca) Euphorbiaceae manutins	Africa	Used as abortifacient, emmenagogue Used for the treatment of anaemia	Leaves, stalks	[82,83]
	Nicaragua	Used for the treatment of belly pain, diarrhoea, fever, headache, hypertension	Leaves	[84]
	Papua New Guinea	Used for the treatment of skin blemishes		
	Colombia, Peru	Used for the treatment of worm infections, wounds		
*Mirabilis jalapa* L. (four o’clock flower) Nyctaginaceae MAPs	Brazil, India, Mexico	Used as diuretic, laxative, tonic Used for the treatment of abscess, boils, bruises, diarrhoea, inflammation, pain, piles, ulcers, urticaria, wounds	Leaves, stems	[85,86,87]
*Momordica balsamina* L. (balsam) Cucurbitaceae balsamin, MbRIP-1, momordin II	Africa	Used as anthelminthic, purgative Used for the treatment of arthritis, burns, chicken pox, diabetes, fever, gonorrhoea, inflammation, jaundice, liver and skin diseases, malaria, measles, menorrhea, rheumatisms, scabies, skin allergies, syphilis, ulcers	Fruits, leaves, roots, seeds	[88]
	Pakistan	Used as wound healer	Leaves	[89]
	India	Used for the treatment of diabetes	Not indicated	[54]
	Mediterranean area	Used as detoxifying, laxative	Fruits	[21,35,36]
*Momordica charantia* L. (bitter melon) Cucurbitaceae charantin, MAP 30, MCLs, momorcharins, momordins	Africa, Central and South America	Used as anthelminthic Used for the treatment of burns, chicken pox, diabetes, dysentery, fever, gonorrhoea, hypertension, inflammation, jaundice, kidney and skin diseases, liver, malaria, measles, menorrhea, rheumatisms, scabies, syphilis, ulcers, womb infection	Fruits, leaves, stems	[18,88,90,91,92]
	India	Used as abortifacient, anthelminthic, anti-mutagenic, anti-oxidants, emmenagogue, galactagogue, laxative, Used for the treatment of boils, burns, chronic fever, diabetes, dysmenorrhea, eczema, gout, jaundice, joint pain, kidney stones, leprosy, leucorrhoea, liver and digestive disorders, malaria, piles, pneumonia, psoriasis, rheumatism, snake poisoning	Fruits, seeds, roots, leaves	[93,94,95]
	Thailand	Used as anthelminthic, anti-oxidant, anti-pyretic, blood tonic, carminative, cholagogue, digestive, laxative	Fruits, leaves	[96]
	Turkey	Used for the treatment of cuts, peptic ulcers, wounds	Fruits	[80,97,98]
*Momordica cochinchinensis* (Lour.) Spreng. (red melon) Cucurbitaceae cochinin B, momorcochins	China	Used as abortifacient, emmenagogue, laxative, mucolytic Used for the treatment of dehydration caused by fever or great heat, diabetes, inflammation, retention of placenta	Fruits, seeds	[23,28,29,99]
*Oryza sativa* L. (rice) Poaceae oryza sativa RIP	India	Used for the treatment of diabetes, leucorrhoea	Seeds	[54,100]
	Palestine, Iran, Mediterranean area	Used for the treatment of diarrhoea, peptic ulcer	Fruits	[21,63,100]
	Thailand	Used for the treatment of wound healing	Not indicated	[101]
*Panax ginseng* C.A.Mey (Chinese ginseng) Araliaceae panaxagin	China, India, Japan, Korea	Used as prophylactic, restorative, tonic Used for the treatment of anorexia, diabetes, haemorrhage, hypodynamia, impotence, insomnia, palpitation, shortness of breath	Berries, leaves, rhizomes, roots	[23,102,103,104]
*Panax quinquefolium* L. (American ginseng) Araliaceae quinqueginsin	China, India, Japan, Korea	Used for the treatment of cancer, diabetes, inflammation, metabolic syndrome	Roots	[23,104,105]
*Phytolacca acinosa Roxb* (Indian poke) Phytolaccaceae PAP‑S1aci	China	Used for the treatment of anasarca, ascites, constipation, reduction of swelling and nodules	Roots	[46]
*Phytolacca americana* L. (pokeweed) Phytolaccaceae PAPs	Africa, China, Japan, Korea	Used as laxative Used for the treatment of ascites, fibroids growth, lymphatic congestion, mastitis, oedema, reduction of swelling and nodules, rheumatism, swellings, wound	Fruits, leaves, roots	[46,106,107]
*Phytolacca dodecandra* L’Hér. (African soapberry) Phytolaccaceae dodecandrins	Africa	Used for the treatment of ascariasis, eczema, gonorrhoea, jaundice, malaria, rabies, rheumatic pain, sore throat, syphilis pruritus, vitiligo	Fruits, roots	[108]
*Pisum sativum* L. (pea) Fabaceae pisavins	Pakistan	Used as anti-spermatogenic	Seeds	[89]
	Africa	Used as abortifacient, purgative Used for the treatment of alopecia, arthritis, body ache, conjunctivitis, cutaneous lesions, dermatoses, fractures, headache, mycosis, neuralgia, rheumatism, scabies, sprain	Fruits, leaves, roots, seeds,	[11,82,109,110,111]
	Ancient Egypt	Used as astringent and for the treatment of skin irritation	Seeds	[37,38,39]
*Ricinus communis* L. (castor bean) Euphorbiaceae ricins	Mediterranean area	Used as aperient, detoxifying, emetic, galactogogue, laxative Used for the treatment of phlegm, sunburn, varicose veins	Leaves, seeds	[21,35,36,112]
	China, India,	Used as anthelmintic, anti-bacterial, aphrodisiac, cathartic, laxative Used for the treatment of anti-fertility, arthritic pain, cancer, chronic wounds, convulsions, diabetes, epilepsy, fever, hair loss, inflammation, rhinitis, skin diseases, swelling, ulcers	Latex, leaves, roots, seeds, stem bark	[112,113,114,115,116,117]
	Iran, Pakistan	Used for the treatment of boils, chronic knee osteoarthritis, gout, liver diseases, rheumatism, sores, wounds	Leaves, seeds	[22,118]
	Caribbean, Brazil	Used as anthelminthic, purgative Used for the treatment of burn, erysipelas, flu, hair loss, stomach aches, womb inflammation, wound healing	Leaves	[18,19]
	Africa	Used as abortifacient, tocolytic Used for the treatment of alopecia, arthritis, conjunctivitis, cutaneous lesions, dermatoses, fracture, inflammation, mycosis, neuralgia, rheumatism, scabies, sprain	Fruits, leaves, roots	[82,109,110]
	Ancient Egypt	Used as abortifacient, laxative Used for the treatment of abscessual illness, baldness, polyuria	Fruits, roots	[37,38,39]
*Sambucus ebulus* L. (dwarf elder) Adoxaceae ebulins, ebulitins, SEA	Iran	Used for the treatment of bee/nettle bites, bone/joint disorders, chronically inflammatory diseases, eczema, fungal skin disorders, haemorrhoids	Aerial parts, fruits, leaves, rhizomes, roots	[119,120,121,122]
	Turkey	Used for the treatment of cold, cough, knee ache, rheumatism pain, snakebites, urticaria,	Flowers, fruits, leafy stems, leaves, roots	[97,123]
*Sambucus nigra* L. (elderberry) Adoxaceae nigrins, nigritins, SNAs, SNLRPs	Mediterranean area	Used as diuretic, laxative Used for the treatment of asthma, burns, cough, eye inflammation, haemorrhoids, pimples, throat infections, scalds, wounds	Bark, buds, flowers, leaves, marrow, pith	[124]
	North Europe	Used as cathartic, diaphoretic in upper respiratory tract infections, diuretic, laxative Used for the treatment of headache, low back or neuropathic pain, toothache	Fruits	[125]
	East Europe	Used as anti-septic, anti-catarrhal, diuretic, expectorant, sedative Used for the treatment of arteriosclerosis, colds, fever, hypertension, inflammations, insomnia, perspiration, nervous tachycardia	Flowers	[126,127]
	Turkey	Used for the treatment of abscesses, asthma, bronchitis, haemorrhoids, prostatitis, rheumatisms	Flowers, fruits, leaves	[97,121,128]
*Saponaria officinalis* L. (soapwort) Caryophyllaceae saporins	Mediterranean area	Used as emmenagogue	Not indicated	[21,35,36]
*Siraitia grosvenorii* (Swingle) C. Jeffrey ex A.M. Lu & Zhi Y. Zhang (monk fruit) Cucurbitaceae momorgrosvin	China, India, Japan, Korea	Used as abortifacient, emmenagogue, laxative, mucolytic Used for the treatment of dehydration caused by fever or great heat, dire thirst, dry cough, pharyngitis, retention of placenta, sore throat sweetener	Fruits, roots	[23,28,29,129,130,131]
*Spinacia oleracea* L. (spinach) Amaranthaceae SoRIPs	India, Turkey	Used as diuretic an for the treatment of diabetes	Aerial parts	[33,54]
*Stellaria media* (L.) Vill. (chickweed) Caryophyllaceae RIP Q3	China, India	Used for the treatment of arthritis, bronchitis, dermatitis, inflammation, mange, period and rheumatic pains, skin diseases, viral infections	Whole plant	[129,132]
*Trichosanthes cucumerina* L. (snake gourd) Cucurbitaceae TCSL	French Guiana, India, Sri lanka,	Used as anthelminthic, cathartic, hepato- and gastro-protector, hypoglycaemic, larvicidal Used for the treatment of boils, bronchitis, cancer, dermatitis, diabetes, fever, eczema, headache, indigestion, inflammation, liver congestion, ovulation, psoriasis, sores, stomach disorders, urticaria, ulcers	Fruits, leaves, roots	[4,133,134,135]
*Trichosanthes cucumeroides* (Ser.) Maxim. (Japanese snake gourd) Cucurbitaceae β-trichosanthin	China	Used as abortifacient, emmenagogue, mucolytic Used for the treatment of dehydration caused by fever or great heat, diabetes, retention of placenta	Fruits, root tubers	[23,136]
*Trichosanthes kirilowii* Maxim. (Chinese snake gourd) Cucurbitaceae kirilowins, TAP 29, TK-35, trichobitacin, trichokirins, trichomislin, trichosanthins, trichosanthrip	China	Used as abortifacient, emmenagogue, galactagogue, laxative, mucolytic Used for the treatment of cancer, cough, dehydration caused by fever or great heat, diabetes, gynaecological illnesses, hydatiform moles, invasive moles, retention of placenta	Fruits, root tubers	[17,23,28,29,137,138,139]
*Triticum aestivum* L. (wheat) Poaceae tritins	Ancient Egypt, India, Mediterranean area	Used as tonic Used for the treatment of burns, gynaecological disorders leucorrhoea, skin lesions, sprains, whitlows, wounds	Seeds	[16,21,35,36,37,38,39,124]
*Viscum album* L. (mistletoe) Santalaceae mistletoe lectins	India	Used for the treatment of diabetes	Leaves	[140]
	Germany, Mediterranean Europe	Used as emetic Used for the treatment of abscesses, cancer, chilblains, gynaecological disorders, scrofula, spleen diseases	Not indicated	[124,141,142]
	Africa	Used for the treatment of asthma, bronchitis, cardio-vascular diseases, hypertension, painful menstruation, stomach ailment, stroke, tumour swellings	Leaves, whole plant	[106,143,144]
	Turkey	Used for the treatment of asthma, atherosclerosis, brain cancer, breathing difficulties, bronchitis, cardiovascular diseases, chronic cramps, diabetes, headache, heart palpitations, hypercholesterolemia, hypertension, hypogalactia, prostatitis, rheumatism, splenopancreatic diseases, sterility, stomach problems, stroke, tachycardia, throat ache, tonic, toothache, tonsillitis	Fruits, leafy, leaves branches, twigs	[97,145,146,147,148]
*Ximenia americana* L. (sea lemon) Oleaceae riproximins	Africa	Used for the treatment of anaemia, arthritis, amenorrhea, anxiety, asthma, back and abdominal pain, bleeding after menstruation, burns, candida infection, conjunctivitis, cough, dermatitis, diabetes, dysentery, dysmenorrhoea, fever, food poisoning, gastric ulcer, gingivitis, headaches, haemorrhoids, infertility, internal wounds, malaria, measles, mental diseases, migraine, otitis, schistosomiasis, sinusitis, snake/scorpion bites, throat infections, tonic, tooth ache, urinary tract infections, uterine cancer, uterine prolapse, vaginitis, varicose veins, vomiting, wounds	Bark, fruits, leaves, roots, seeds, stems	[149,150,151]
	Brazil	Used as laxative Used for the treatment of cough, diabetes, hoarseness, inflammation, obesity, osteoporosis pain, venereal disease, wound healing	Bark, fruits, roots	[19]
*Zea mays* L. (corn) Poaceae b-32	China, India, Japan, Korea, Middle Est	Used as digestive Used for the treatment of cancer, diabetes, different diseases (circulatory, connective tissue, genitourinary, musculoskeletal, respiratory system)	Flowers	[23,152]
	Africa	Used for the treatment of inflammation, pain	Husk	[153]
	Peru	Used as anti-oxidant, tonic Used for the treatment of amenorrhea, dysentery, dysmenorrhea, flu, obesity, oliguria	Seeds	[18,154]
